# Criterion Validity of Wrist-Worn IMU-Derived Smoothness Metrics During Functional Upper-Limb Tasks

**DOI:** 10.3390/bioengineering13101233

**Published:** 2026-10-21

**Authors:** Serena Cerfoglio, Lucia Donno, Mattia Lovato, Carlalberto Francia, Manuela Galli, Veronica Cimolin

**Affiliations:** 1 Department of Electronics, Information and Bioengineering, Politecnico di Milano, Piazza Leonardo da Vinci 32, 20133 Milano, Italy; serena.cerfoglio@polimi.it (S.C.); mattia.lovato@polimi.it (M.L.); carlalberto.francia@polimi.it (C.F.); manuela.galli@polimi.it (M.G.); veronica.cimolin@polimi.it (V.C.); 2 IRCCS Istituto Auxologico Italiano, San Giuseppe Hospital, Strada Luigi Cadorna 90, 28824 Piancavallo, Italy; 3 E4Sport-Human Performance Lab, Polo Territoriale di Lecco, Politecnico di Milano, Via G. Previati 1/c, 23900 Lecco, Italy

**Keywords:** inertial measurement units, upper-limb kinematics, movement smoothness, criterion validity, optical motion capture, functional motor tasks

## Abstract

Wearable inertial measurement units (IMUs) may facilitate quantitative assessment of upper-limb motor function outside laboratory settings; however, the validity of IMU-derived movement smoothness metrics compared with optical motion capture remains unclear. This study aimed to investigate the criterion validity of wrist-worn IMU-derived smoothness metrics during functional upper-limb tasks. Twenty-seven healthy adults (14 females, 13 males) performed two functional tasks, Reaching and Hand-to-mouth, with both upper limbs. Movements were simultaneously recorded using a marker-based motion capture system and two wrist-worn IMUs, both sampling at 100 Hz. Acceleration profiles were obtained from MoCap-derived wrist trajectories and IMU signals, and four smoothness metrics were calculated: Spectral Arc Length (SPARC), normalized root mean square (nRMS), Normalized Jerk (NJ), and Log Dimensionless Jerk (LDLJ). Criterion validity was assessed through Pearson correlation, estimation error (RMSE and MAE), Bland–Altman analysis, and proportional bias assessment. During the Reaching task, IMU-derived metrics showed significant correlations with MoCap values for all indices (r = 0.41–0.74), with NJ showing the strongest association. SPARC showed the smallest mean bias between systems. During the Hand-to-mouth task, significant correlations were observed only for SPARC and NJ (r = 0.39–0.47), with increased discrepancies for nRMS and LDLJ. Wrist-worn IMUs provide comparable smoothness estimates to MoCap during specific upper-limb tasks, but agreement depends on both the selected metric and movement characteristics. These findings highlight the need for task- and metric-specific validation before translating laboratory-based movement quality assessment into wearable settings.

## 1. Introduction

Upper-limb motor function is essential for the performance of activities of daily living, making its accurate assessment an important component of clinical evaluation and rehabilitation [1]. Standardized clinical scales and functional tests provide valuable information on motor impairment and activity limitations, but may offer limited insight into how a movement is actually performed. Instrumented kinematic assessment is not part of these clinical tools, but can complement them by providing objective and continuous measures of movement quality and by helping distinguish improvements related to restitution of motor control from those achieved through compensatory strategies [2,3,4].

Among the different characteristics of movement quality, smoothness has received particular attention as a potential quantitative marker of motor control and recovery. Healthy and well-coordinated movements are generally characterized by continuous motion profiles, whereas motor impairments result in more intermittent and segmented movements [5,6]. In individuals after stroke, the population in which upper-limb smoothness has been most extensively investigated, smoothness has been shown to improve during recovery and to provide complementary information on upper-limb motor performance [7,8,9].

Despite its potential clinical relevance, movement smoothness can be quantified using several different metrics, which vary in their mathematical formulation and sensitivity to movement characteristics. A systematic review identified 32 smoothness measures used for upper-limb reaching after stroke, highlighting the considerable methodological heterogeneity in this field [4]. Among the most widely investigated approaches, jerk-based measures quantify fluctuations in movement through higher-order derivatives of kinematic signals, whereas spectral measures characterize smoothness from the frequency content of the movement profile [6]. Log Dimensionless Jerk (LDLJ) and Spectral Arc Length (SPARC) have received particular attention because of their desirable mathematical properties and application to upper-limb movements [4,6]. However, they capture different aspects of movement and cannot be considered interchangeable. In healthy participants, temporal-domain metrics including LDLJ were associated with movement duration, whereas SPARC showed lower intra- and inter-subject variability and lower dependence on movement time [10]. Similarly, in individuals with subacute stroke, both SPARC and LDLJ showed high reliability, but differed in their sensitivity to specific movement characteristics [11]. Thus, the choice of metric may substantially influence the resulting characterization of movement smoothness.

A further methodological issue concerns the technology used to acquire movement data. Optical motion capture (MoCap) provides detailed three-dimensional reconstruction of movement and is widely used as a reference approach for upper-limb kinematic analysis. However, its cost, infrastructure requirements and restricted acquisition environment limit its applicability for repeated assessments and routine clinical use [2,12]. Wearable inertial measurement units (IMUs) offer a more portable alternative and facilitate quantitative assessment in clinical and real-world environments [12,13]. Nevertheless, translating smoothness metrics from laboratory-based motion capture to inertial sensing is not straightforward, since the two technologies provide different representations of movement. While MoCap reconstructs spatial trajectories, IMUs directly measure quantities such as linear acceleration and angular velocity. Consequently, smoothness metrics developed for a specific kinematic signal cannot necessarily be transferred unchanged to another signal.

This issue has been specifically investigated for smoothness assessment. Melendez-Calderon et al. demonstrated that smoothness estimates obtained from inertial sensors depend on both the selected metric and the kinematic quantity from which they are derived, and proposed an acceleration-based formulation of LDLJ suitable for discrete translational movements measured using IMUs [14]. Since two measures may be highly correlated and still differ systematically, validation studies increasingly assess the agreement and interchangeability between systems, for instance through Bland–Altman analysis and intraclass or concordance correlation coefficients, rather than relying on correlation alone [15,16]. More recent studies comparing IMU- and MoCap-derived upper-limb measures have provided encouraging results, while also showing that agreement varies according to the parameter considered. Using a wrist-worn IMU in healthy adults, Hughes et al. reported high agreement with optical motion capture for SPARC, movement time and peak velocity during functional tasks (ICC = 0.891–0.995), with no significant systematic errors in the Bland–Altman analysis [17]. Merlau et al. used Bland–Altman analysis and ICC and reported parameter-dependent agreement between the two technologies during reaching and grasping tasks after stroke, with the lowest agreement for the number of sub-movements and a strong influence of calibration and sensor-fusion procedures [18], while Unger et al. found limits of agreement below the minimal clinically important difference for most movement-quality measures during a functional drinking task, including acceleration-based LDLJ [19]. However, evidence remains limited regarding whether different smoothness metrics provide comparable information when derived from wearable and laboratory-based measurements. This is particularly relevant because agreement demonstrated for one smoothness metric cannot necessarily be generalized to another, given their different mathematical properties and sensitivity to movement characteristics [10,11].

Therefore, the present study aimed to compare upper-limb movement smoothness obtained from wearable inertial sensing and optical motion capture during two functional tasks, Reaching and Hand-to-mouth, in a sample of healthy adults. Specific movement smoothness metrics were considered, selected to represent different approaches to smoothness quantification: SPARC, representing a spectral-based approach; NJ and LDLJ, representing jerk-based approaches; and nRMS, representing an acceleration amplitude-based approach. The study aimed to determine the extent to which wearable-derived measures provide information comparable to laboratory-based motion analysis and whether agreement between systems is influenced by the selected smoothness metric and motor task. As a first step, the validation was conducted in healthy participants in order to characterize the agreement between the two systems under controlled conditions, separating measurement-related differences from the variability associated with pathological movement. This represents a necessary premise before extending the assessment to clinical populations.

## 2. Materials and Methods

### 2.1. Participants

Data collection was conducted between October and December 2025 at the “Luigi Divieti” Posture and Movement Analysis Laboratory of Politecnico di Milano (Milan, Italy). Twenty-seven healthy adults (14 females and 13 males; age: 26.33 ± 1.63 years; height: 171.81 ± 7.57 cm; body mass: 65.15 ± 12.10 kg; BMI: 21.91 ± 2.91 kg/m^2^) were enrolled in the study on a voluntary basis. Participants were included if they had a BMI within the normal range (18.5–24.9 kg/m^2^) and they reported no musculoskeletal conditions potentially affecting upper-limb motor performance. All participants were right-handed.

The study protocol was approved by the Ethics Committee of Politecnico di Milano (protocol n. 49/2025, 23 June 2025) and was conducted in accordance with the principles of the Declaration of Helsinki (1964) and its subsequent amendments. Before enrollment, all participants received detailed information regarding the study procedures and their right to withdraw at any time. Written informed consent was obtained prior to data collection.

### 2.2. Experimental Setup and Testing Protocol

Upper-limb movements were recorded using an eight-camera marker-based optoelectronic system (SMART DX 400, BTS Bioengineering S.p.A., Milan, Italy), hereafter referred to as MoCap, with a sampling frequency of 100 Hz. A passive reflective marker was positioned bilaterally on the wrists to reconstruct the three-dimensional trajectories of the wrist markers during task execution.

Simultaneously, participants were equipped with two IMUs (AX6, Axivity Ltd., Newcastle upon Tyne, UK) integrating a triaxial accelerometer and a triaxial gyroscope, with configurable ranges. In the present study, the accelerometer range was set to ±8 g and the gyroscope range to ±2000°/s. Data were acquired at a sampling frequency of 100 Hz. The IMUs were positioned bilaterally on the wrists and secured using elastic straps to minimize relative displacement between the device and the underlying body segment. The IMUs recorded tri-axial linear acceleration and angular velocity during task execution.

Two functional upper-limb motor tasks were considered, namely Reaching and Hand-to-mouth. Each task was performed separately with the right and left upper limbs at a natural, self-selected movement speed.

**Figure 1:**
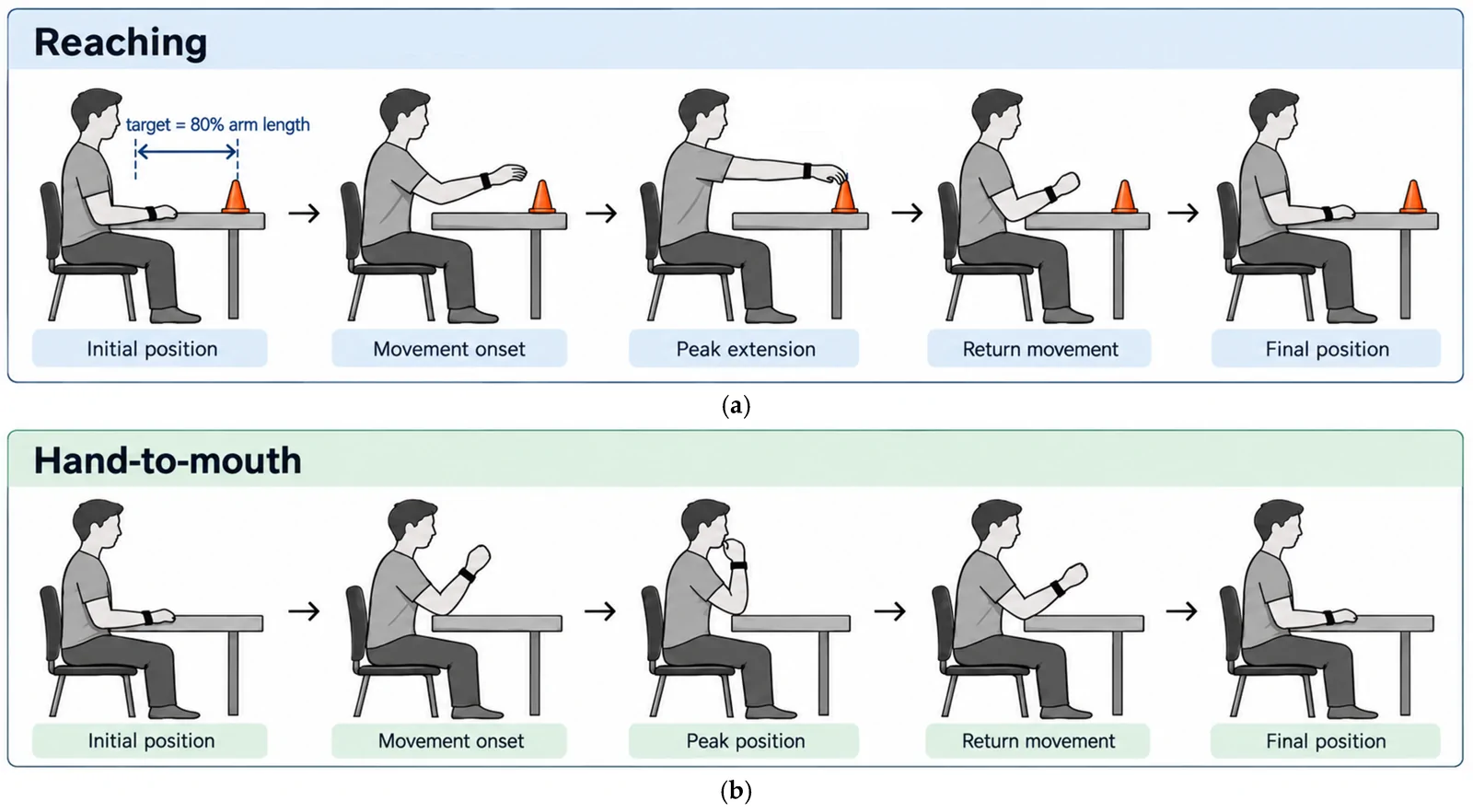
Schematic representation of the upper-limb motor tasks included in the experimental protocol: (**a**) Reaching task and (**b**) Hand-to-mouth task.

During the Reaching task, participants were seated with both wrists resting on a table in a standardized initial position (Figure 1a). Participants were instructed to reach forward and touch a target positioned at 80% of their arm length, and then return the hand to the starting position. During the Hand-to-mouth task, participants started from the same seated initial position and were instructed to bring the hand to the mouth and subsequently return it to the starting position (Figure 1b).

For both tasks, twelve repetitions per side were performed, consisting of two series of six repetitions each.

### 2.3. Signal Processing

#### 2.3.1. Data Synchronization

Since the optical motion capture and IMU systems were not hardware-synchronized, temporal alignment was performed offline before subsequent processing.

At the beginning of each acquisition, participants performed a dedicated synchronization maneuver consisting of at least three consecutive taps of the instrumented hand on the table. The resulting sequence of peaks was identified in both recordings and used to estimate the temporal offset between systems.

The magnitude of the three-dimensional wrist-marker velocity obtained from the MoCap system and the magnitude of the IMU gyroscope signal were used as reference signals. The residual temporal lag was estimated through normalized cross-correlation, and the optimal lag was defined as the temporal shift corresponding to the maximum correlation between the two signals. The final alignment was visually inspected, and the synchronized recordings were cropped to their common temporal interval before further processing.

#### 2.3.2. Movement Segmentation

**Figure 2:**
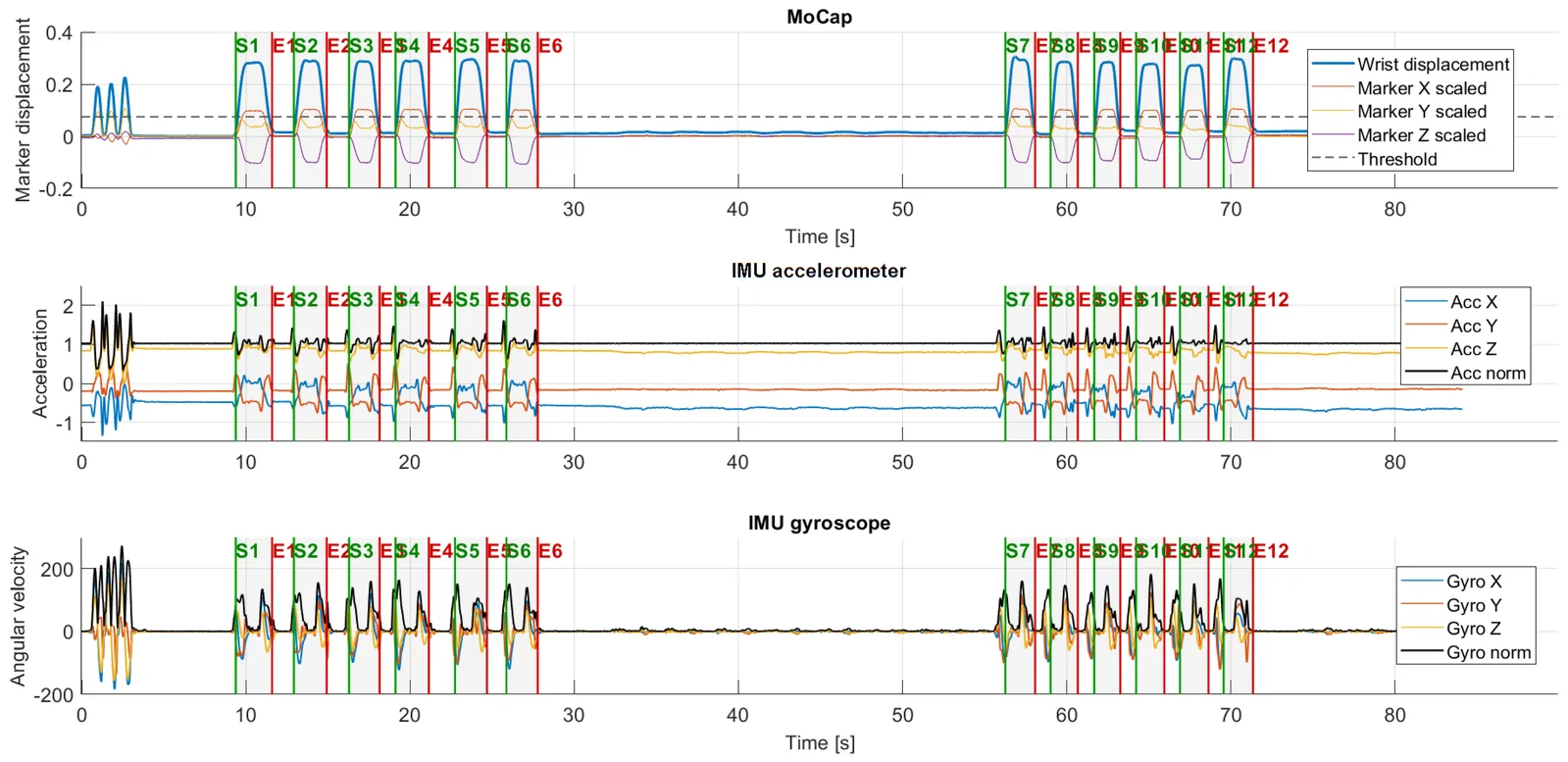
Example of movement segmentation procedure. Representative MoCap and IMU signals showing the identification of upper-limb movement repetitions. The initial peaks (unnumbered segment on the left) correspond to the synchronization maneuver and were excluded from the analysis. Repetition boundaries were identified from the MoCap-derived wrist displacement profile and transferred to the synchronized IMU recordings. Green and red vertical lines indicate movement onset and offset, respectively.

Movement repetitions were segmented from MoCap data, and the identified temporal boundaries were subsequently applied to the synchronized IMU recordings. This approach ensured that all parameters were calculated over identical temporal intervals for both measurement systems (Figure 2).

The three-dimensional wrist-marker trajectory was low-pass filtered using a fourth-order zero-phase Butterworth filter with a cut-off frequency of 6 Hz [20]. A reference wrist position was calculated as the median marker position during the initial static phase. Wrist displacement from the reference position was calculated as (Equation (1)):

(1) $$d\left(t\right)=∥p\left(t\right)-p_{0}∥$$

where d(t) represents wrist displacement at time t, p(t) is the three-dimensional wrist-marker position, and $p_{0}$ is the reference position.

The displacement profile was smoothed using a 0.10 s moving-average window. Movement onset and offset were automatically identified using an adaptive thresholding procedure based on displacement variability. Short interruptions within the same movement were automatically closed, while segments outside predefined duration criteria were excluded.

### 2.4. Smoothness Metrics

For each identified repetition, corresponding MoCap and IMU segments were extracted using the same temporal boundaries. Signals were resampled onto a common temporal vector at 100 Hz using linear interpolation.

For the MoCap system, acceleration was reconstructed from the filtered wrist-marker trajectory through numerical differentiation. For the IMU system, acceleration was directly obtained from the triaxial accelerometer signals. The same filter was applied to both systems, so that differences between them would not depend on different spectral content of the signals; this cut-off preserves the frequency content of voluntary upper-limb movements [20]. Since sensor orientation was not considered in the present analysis, the static acceleration component was attenuated by subtracting the median acceleration vector calculated within each repetition. This follows the approach proposed by Melendez-Calderon et al. for acceleration-based smoothness estimation in discrete movements, in which removing the constant offset of the accelerometer signal prevents the overestimation of the acceleration amplitude used for normalization [14]. The median was used instead of the mean to limit the influence of movement-related acceleration peaks on the offset estimate. This correction was applied to IMU signals only, as MoCap-derived acceleration does not include a gravitational component.

For both measurement systems, the acceleration magnitude was calculated as (Equation (2)):

(2) $$a_{mag}\left(t\right)=\sqrt{a_{x}{\left(t\right)}^{2}+a_{y}{\left(t\right)}^{2}+a_{z}{\left(t\right)}^{2}}$$

where $a_{mag}\left(t\right)$ represents the acceleration magnitude, and $a_{x}\left(t\right)$, $a_{y}\left(t\right)$, and $a_{z}\left(t\right)$ are the acceleration components along the three axes.

Although acceleration was obtained through different approaches, namely numerical differentiation of wrist-marker trajectories for MoCap and direct measurement from the accelerometer for IMU, the same acceleration-magnitude profile was used for the calculation of all movement smoothness metrics, allowing direct comparison between measurement systems. The acceleration magnitude is independent of sensor orientation, and therefore allows a direct comparison between IMU and MoCap signals expressed in different reference frames.

Movement smoothness was quantified using four acceleration-derived metrics: Spectral Arc Length (SPARC), normalized root mean square (nRMS), Normalized Jerk (NJ), and Log Dimensionless Jerk (LDLJ). All metrics were calculated independently for each valid repetition and for each measurement system.

#### 2.4.1. Spectral Arc Length (SPARC)

SPARC was used to characterize the frequency-domain complexity of the acceleration profile. For each repetition, the acceleration-magnitude signal was normalized and transformed into the frequency domain using Fast Fourier Transform (FFT) [6].

Only frequency components up to 10 Hz were retained, and the spectrum was truncated using a normalized amplitude threshold of 0.05. SPARC was calculated as the negative arc length of the normalized frequency spectrum (Equation (3)):

(3) $$SPARC=-\sum_{i=1}^{N-1}\sqrt{{\left(f_{i+1}-f_{i}\right)}^{2}+{\left(A_{i+1}-A_{i}\right)}^{2}}$$

where $f_{i}$ represents normalized frequency and $A_{i}$ the corresponding normalized spectral amplitude. Values closer to zero indicate smoother movement profiles.

#### 2.4.2. Normalized Root Mean Square (nRMS)

nRMS was calculated to quantify the amplitude of acceleration fluctuations during movement. The metric was defined as (Equation (4)):

(4) $$nRMS=\frac{\sqrt{\frac{1}{N}\sum_{i=1}^{N}{\left(a_{mag}\left(i\right)-{\bar{a}}_{mag}\right)}^{2}}}{A}$$

where $a_{mag}$ represents the acceleration magnitude, ${\bar{a}}_{mag}$ its mean value within the repetition, N the number of samples, and A the peak-to-peak range of the acceleration magnitude within the repetition ($A=max\left(a_{mag}\right)-min\left(a_{mag}\right)$). Normalization by the peak-to-peak range makes nRMS dimensionless and independent of movement intensity and of the measurement units of each system.

#### 2.4.3. Normalized Jerk (NJ)

NJ was calculated as a time-domain measure of movement smoothness based on the ratio between the root mean square of the jerk magnitude and that of the acceleration magnitude, scaled by movement duration, adapted from [21] (Equation (5)):

(5) $$NJ=T\cdot \frac{\sqrt{\frac{1}{N}\sum_{i=1}^{N}∥j\left(i\right){∥}^{2}}}{\sqrt{\frac{1}{N}\sum_{i=1}^{N}a_{mag}{\left(i\right)}^{2}}}$$

where T represents movement duration, N the number of samples, ∥j(i)∥ the magnitude of the jerk vector j(t)=da(t)/dt, obtained by differentiating the three acceleration components, and $a_{mag}$ the acceleration magnitude. NJ is dimensionless, and higher values indicate less smooth movements.

#### 2.4.4. Log Dimensionless Jerk (LDLJ)

LDLJ was calculated as a complementary dimensionless measure of smoothness, accounting for movement duration and acceleration amplitude [22] (Equation (6)):

(6) $$LDLJ=-\ln\left(\frac{T}{A^{2}}\int_{0}^{T}{\left(\frac{da_{mag}\left(t\right)}{dt}\right)}^{2}dt\right)$$

where T represents movement duration and A the acceleration-magnitude range.

### 2.5. Statistical Analysis

Statistical analysis was performed separately for the Reaching and Hand-to-mouth tasks using JASP (Version 0.97; JASP Team, Amsterdam, The Netherlands) and custom scripts in Matlab (R2025a, MathWorks, Natick, MA, USA).

For each task, participants performed 12 repetitions with each upper limb. The first and last repetitions were excluded to minimize potential effects of task start and end, and the value used for each metric and each limb was calculated as the mean of the remaining 10 repetitions. Both limbs of each participant were included in the analysis, resulting in two observations per participant. To account for the dependence between limbs of the same participant, all 95% confidence intervals (CI) and *p*-values were obtained through a participant-level cluster bootstrap with 10,000 resamples, in which participants were resampled with replacement together with both of their limbs. The same resamples were used for both tasks and all metrics, so that all comparisons were paired. Confidence intervals were computed with the percentile method, and two-sided *p*-values were derived from the bootstrap distributions.

Criterion validity of the IMU-derived movement quality metrics against the MoCap reference was evaluated separately for SPARC, nRMS, NJ, and LDLJ. Before correlation analysis, the normality of the IMU- and MoCap-derived values for each metric was assessed using the Shapiro–Wilk test (*p* > 0.05 indicating no significant deviation from normality), while the linearity of the relationship between the two measurement systems was verified by visual inspection of MoCap vs. IMU scatterplots. As both assumptions were satisfied, the linear relationship between IMU- and MoCap-derived values was assessed using Pearson’s correlation coefficient (*r*), together with its 95% confidence interval (CI) and associated *p*-value. This index was used to quantify the strength of the association between systems, i.e., the extent to which IMU-derived metrics rank participants consistently with the reference. Since a strong correlation does not imply that two methods provide the same values, agreement between systems was assessed separately, as described below [15,16].

Agreement between IMU- and MoCap-derived values was further assessed using Bland–Altman analysis and, for each metric, paired differences were calculated as MoCap—IMU. This analysis quantifies the systematic difference between systems through the mean bias and the expected range of individual differences through the 95% limits of agreement, which were computed as the mean bias ± 1.96 SD of the differences. Finally, proportional bias was assessed by linear regression of the MoCap—IMU differences against the mean of the paired MoCap and IMU measurements. The regression slope (B) was reported together with its 95% CI and associated *p*-value. A slope significantly different from zero (*p* < 0.05) was considered indicative of proportional bias.

The magnitude of the estimation error between the two measurement systems was quantified using the root mean square error (RMSE) and mean absolute error (MAE).

To compare the measurement error across metrics with different scales and across tasks, a normalized MAE (nMAE) was computed as the MAE divided by the between-subject standard deviation of the MoCap values. An nMAE of 1 indicates that the average IMU error equals the typical between-subject variability measured by the reference system. Differences in nMAE between tasks (Hand-to-mouth minus Reaching) were tested for each metric, and differences between all pairs of metrics were tested within each task. *p*-values for these comparisons were adjusted using the Holm method within each family of comparisons.

Statistical significance was set at *p* < 0.05 for all analyses.

## 3. Results

Criterion validity of the IMU-derived metrics against the MoCap reference was assessed separately for the Reaching and Hand-to-mouth tasks in 27 healthy participants. For each task, measurements obtained from the right and left upper limbs were pooled, obtaining a total of 54 limb-level observations. The analysis focused on four movement quality metrics: SPARC, nRMS, NJ, and LDLJ. For each metric, the relationship and agreement between IMU- and MoCap-derived values were characterized using Pearson correlation coefficients, estimation errors expressed as root mean square error (RMSE) and mean absolute error (MAE), Bland–Altman analysis, and assessment of proportional bias.

### 3.1. Reaching Task

Table 1 summarizes the relationship between IMU- and MoCap-derived metrics during the Reaching task. Pearson correlation coefficients were used to quantify the linear association between the two measurement systems, whereas RMSE and MAE were used to quantify the magnitude of the estimation error.

All four indices showed significant positive linear correlations between IMU and MoCap. The largest correlation coefficient was observed for NJ (r = 0.741, 95% CI [0.498, 0.885], *p* < 0.001). This was followed by LDLJ (r = 0.505, 95% CI [0.315, 0.672], *p* < 0.001) and SPARC (r = 0.491, 95% CI [0.193, 0.668], *p* < 0.001). The nRMS index showed the smallest, although still statistically significant, correlation coefficient (r = 0.412, 95% CI [0.125, 0.620], *p* = 0.002).

The magnitude of the discrepancy between measurements was additionally quantified by RMSE and MAE. For SPARC, RMSE and MAE were 0.4121 and 0.3252, respectively, whereas values of 0.0454 and 0.0388 were obtained for nRMS. Finally, NJ showed RMSE equal to 3.8580 and MAE of 3.4092, while the corresponding values for LDLJ were 0.5874 and 0.4851, respectively.

To quantify the agreement between the two measurement methods, Bland–Altman analysis was additionally performed. Table 2 reports mean difference between MoCap and IMU measurements (mean bias), its 95% CI, the 95% limits of agreement (LoA), and the regression analysis used to assess the proportional bias. Considering that differences were calculated as MoCap-IMU, a positive bias indicates that, on average, IMU generated lower values than MoCap; instead, a negative bias indicates higher IMU values than MoCap. The associated *p*-value (*p*) tests whether the slope significantly differs from zero; *p* < 0.05 indicates the presence of a significant proportional bias, whereas *p* ≥ 0.05 indicates no evidence of proportional bias.

The Bland–Altman analysis identified different patterns of systematic difference across the four metrics. For SPARC, the mean bias was close to zero (0.046, 95% CI [−0.032, 0.128]), with the confidence interval including zero. In contrast, the confidence intervals of the mean biases for nRMS, NJ, and LDLJ did not include zero. nRMS showed a negative mean bias of −0.037 (95% CI [−0.045, −0.029]), indicating higher IMU-derived values on average, whereas NJ showed a positive mean bias of 3.384 (95% CI [2.777, 4.078]), indicating lower IMU-derived values. LDLJ showed a negative mean bias of −0.482 (95% CI [−0.592, −0.375]), corresponding to higher IMU-derived values on average.

Regression of the MoCap—IMU differences against the mean of the paired measurements identified significant proportional bias for SPARC, NJ, and LDLJ. For SPARC, the regression slope was negative (B = −0.329, 95% CI [−0.713, −0.116], *p* < 0.001), indicating that the difference between MoCap and IMU decreased as the magnitude of the measurements increased. A significant positive slope was found for NJ (B = 0.328, 95% CI [0.064, 0.521], *p* = 0.013), indicating an increase in the MoCap—IMU difference at higher measurement values. LDLJ also showed a significant negative slope (B = −0.546, 95% CI [−0.816, −0.220], *p* < 0.001), with increasingly negative MoCap—IMU differences as measurement magnitude increased. In contrast, no significant proportional bias was detected for nRMS (B = 0.208, 95% CI [−0.185, 0.559], *p* = 0.262).

**Figure 3:**
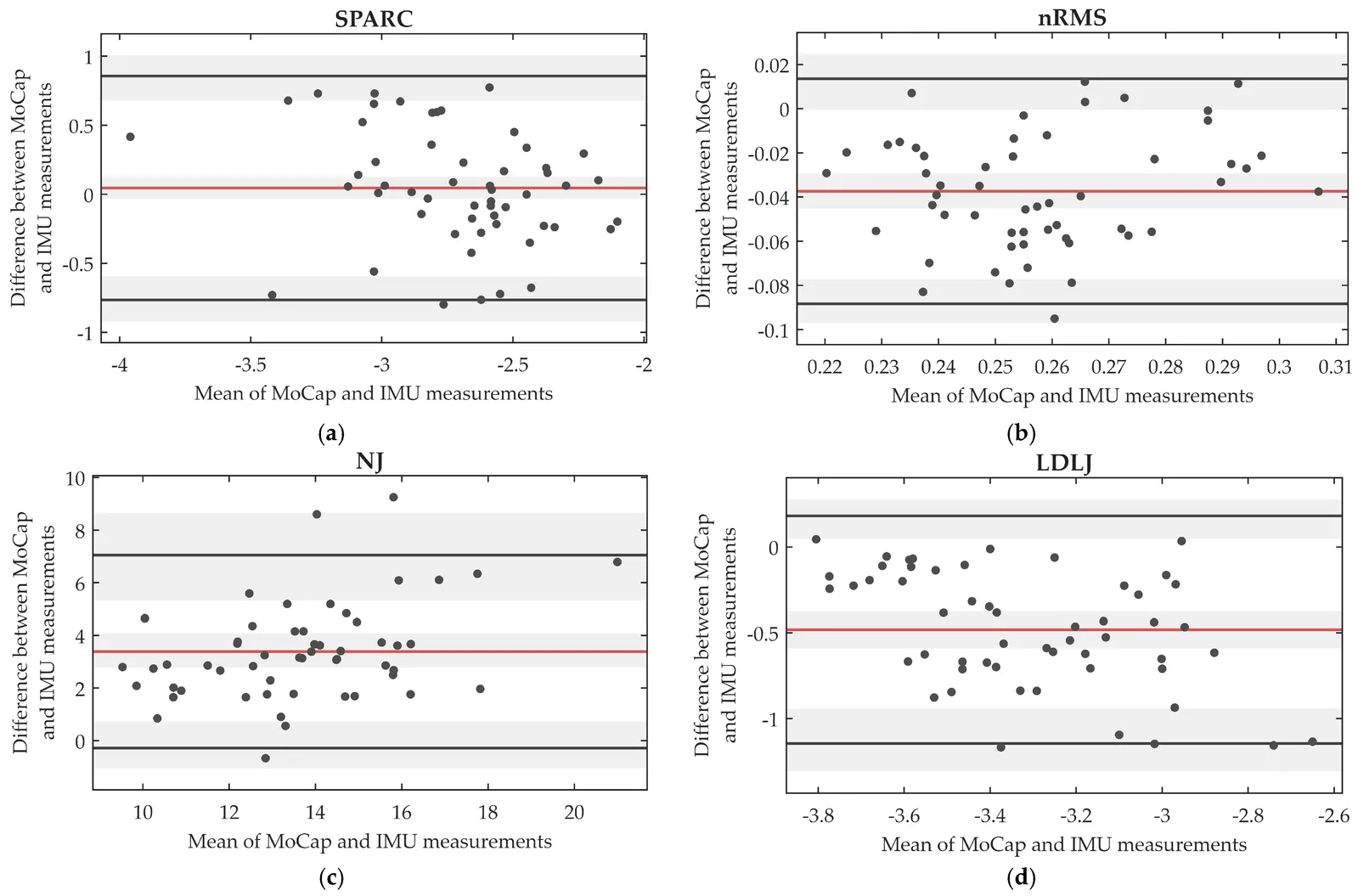
Bland–Altman plots comparing indices derived from IMU and MoCap during the Reaching task: (**a**) SPARC, (**b**) nRMS, (**c**) NJ, (**d**) LDLJ. The red line indicates the mean bias, the black lines indicate the upper and lower 95% LoA, and the gray shaded areas represent the confidence intervals of the LoA.

Figure 3 depicts the Bland–Altman plots for the four indices during the reaching task, showing the distribution of paired differences across the measurement range together with the mean bias and limits of agreement. In each subplot, the horizontal axis represents the mean of the two measurements, and the vertical axis represents their difference (MoCap—IMU). Each point represents a paired observation. Red and black lines indicate the mean bias and the upper and lower 95% limits of agreement (mean bias ± 1.96 SD); shaded gray areas represent their respective 95% confidence intervals. Positive differences indicate lower IMU values relative to MoCap, whereas negative differences indicate higher IMU values.

For SPARC (Figure 3a), the mean bias was 0.046, with a 95% confidence interval of [−0.032, 0.128], which included zero. The 95% limits of agreement extended from −0.764 to 0.856, showing that individual MoCap—IMU differences occurred in both directions despite the small average difference between the two methods.

Instead, nRMS showed a negative mean bias of −0.037 (95% CI [−0.045, −0.029]), indicating that IMU-derived values were higher than MoCap-derived values on average. The 95% limits of agreement ranged from −0.088 to 0.014 and therefore included differences in both directions despite the negative average bias (Figure 3b). From the measurements obtained for NJ (Figure 3c), a positive mean bias of 3.384 (95% CI [2.777, 4.078]) was observed, indicating lower values obtained from the IMU relative to MoCap.

For LDLJ (Figure 3d), the mean bias was negative and equal to −0.482, indicating higher IMU-derived values on average. The 95% limits of agreement ranged from −1.146 to 0.182 and included zero. Thus, although a systematic negative mean difference was present, individual paired differences could occur in either direction.

### 3.2. Hand-to-Mouth Task

Table 3 summarizes the correlation and estimation error between IMU- and MoCap-derived metrics during the Hand-to-mouth task.

Significant positive linear correlations between IMU and MoCap were observed for SPARC (r = 0.466, 95% CI [0.192, 0.663], *p* < 0.001) and NJ (r = 0.390, 95% CI [0.107, 0.613], *p* = 0.006). In contrast, the correlation for nRMS was not statistically significant (r = 0.150, 95% CI [−0.142, 0.408], *p* = 0.278), with its confidence interval including zero. Similarly, LDLJ showed a correlation coefficient close to zero (r = −0.047, 95% CI [−0.471, 0.328], *p* = 0.740), providing no evidence of a linear relationship between the two measurement systems for this index.

As regards the estimation error, SPARC showed an RMSE of 0.4347 and MAE equal to 0.3623, whereas the corresponding values for nRMS were 0.0508 and 0.0434. For NJ, RMSE and MAE were 7.1757 and 6.9043, respectively, while the corresponding values for LDLJ were 1.0924 and 1.0048.

Bland–Altman agreement and proportional bias for the Hand-to-mouth task are reported in Table 4. As for the Reaching task, differences were calculated as MoCap—IMU, so that positive values indicate lower measurements obtained with the IMU and negative values indicate higher measurements obtained with the IMU relative to MoCap.

The Bland–Altman analysis showed mean biases whose 95% confidence intervals excluded zero for all four metrics, indicating systematic differences between IMU- and MoCap-derived measurements. SPARC showed a positive mean bias of 0.233, corresponding to lower IMU-derived values. Conversely, nRMS showed a negative mean bias of −0.041, indicating higher IMU-derived values. NJ showed a positive mean bias of 6.904, whereas LDLJ showed a negative mean bias of −1.005, indicating higher and lower IMU-derived values, respectively.

Proportional bias was statistically significant for SPARC (B = −0.328, 95% CI [−0.648, 0.037], *p* = 0.028) and LDLJ (B = −1.244, 95% CI [−1.903, −0.854], *p* < 0.001). The negative slopes indicated that the MoCap—IMU difference decreased as the magnitude of the measurements increased. No significant proportional bias was detected for nRMS (*p* = 0.052) and NJ (*p* = 0.600).

**Figure 4:**
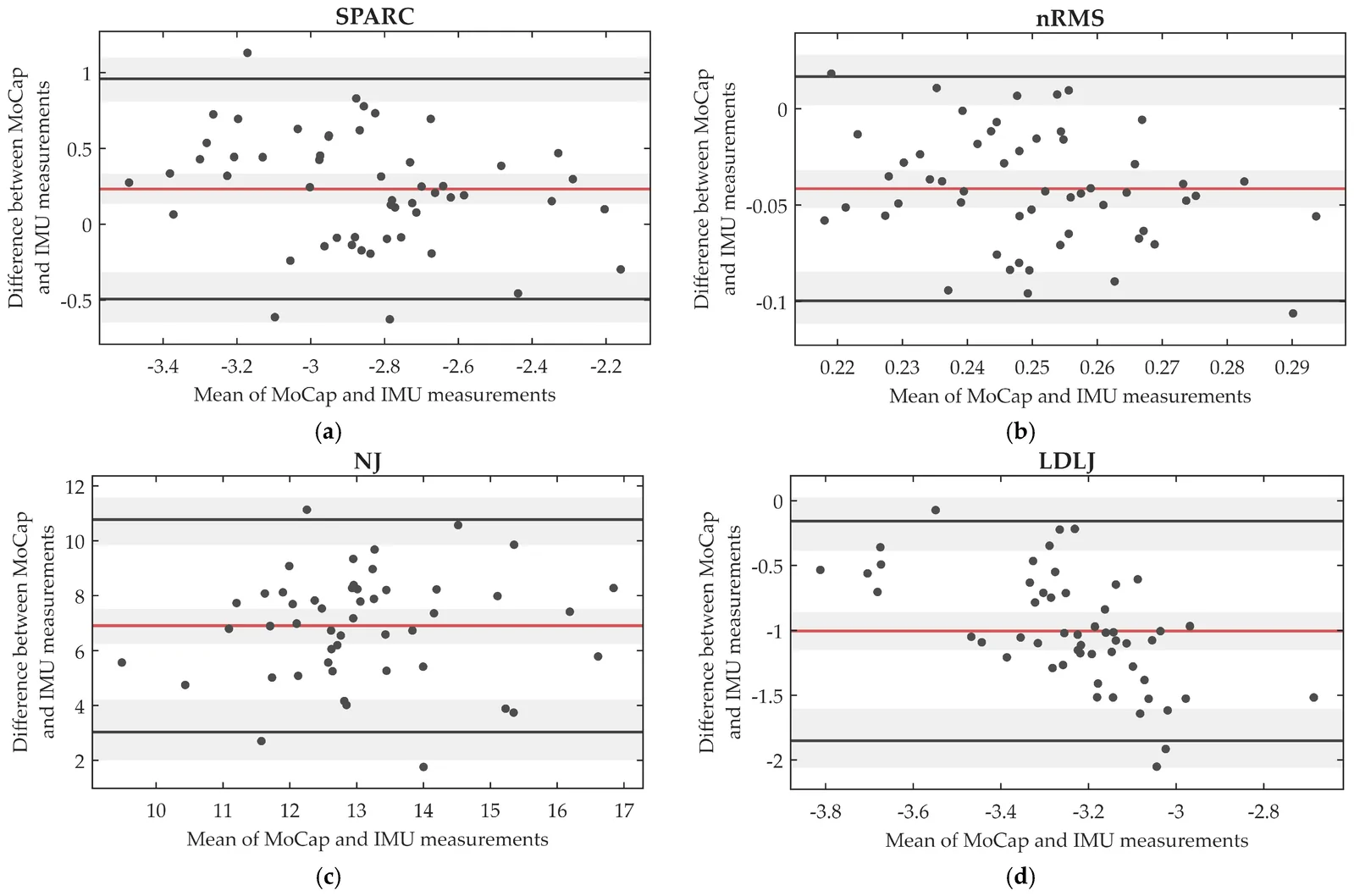
Bland–Altman plots comparing indices derived from IMU and MoCap during the Hand-to-mouth task: (**a**) SPARC, (**b**) nRMS, (**c**) NJ, (**d**) LDLJ. The red line indicates the mean bias, the black lines indicate the upper and lower 95% LoA, and the gray shaded areas represent the confidence intervals of the LoA.

Figure 4 illustrates the Bland–Altman agreement between the two measurement systems for each metric during the Hand-to-mouth task, allowing the magnitude and direction of the differences to be examined across the measurement range.

The Bland–Altman results showed non-zero mean biases for all four indices, with each 95% confidence interval excluding zero. SPARC had a positive mean bias of 0.233 (95% CI [0.134, 0.333]) and limits of agreement of [−0.494, 0.959], indicating lower IMU values on average (Figure 4a). For nRMS, represented in Figure 4b, the mean bias was −0.041 (95% CI [−0.051, −0.032]), with limits of agreement of [−0.100, 0.017], indicating higher IMU values on average. Thus, for both SPARC and nRMS, a systematic average difference was observed even though the limits of agreement included individual differences in both directions.

NJ showed a positive mean bias of 6.904 (95% CI [6.245, 7.512]). Both limits of agreement were entirely positive, ranging from 3.033 to 10.775, indicating that MoCap-derived NJ values were higher than IMU-derived values across the range defined by the limits of agreement (Figure 4c). As depicted in Figure 4d, LDLJ showed the opposite direction of the difference, with a mean bias of −1.005 (95% CI [−1.152, −0.862]) and entirely negative limits of agreement [−1.853, −0.157], indicating higher LDLJ values derived from the IMU relative to MoCap. In addition, the significant negative regression slope (B = −1.244, 95% CI [−1.903, −0.854], *p* < 0.001) showed that this difference became increasingly negative as the magnitude of the measurements increased.

### 3.3. Comparison Between Metrics and Tasks

The nMAE values for each metric and task are reported in Table 5. In both tasks, SPARC showed the lowest nMAE (Reaching: 0.93; Hand-to-mouth: 1.17), whereas LDLJ showed the highest (Reaching: 1.92; Hand-to-mouth: 5.28). All nMAE values were close to or above 1, indicating that the average IMU error was comparable to or larger than the between-subject variability measured by MoCap.

Compared with Reaching, the nMAE during Hand-to-mouth increased significantly for NJ (difference 2.50, *p* < 0.001) and LDLJ (difference 3.37, *p* < 0.001), whereas no significant differences between tasks were observed for SPARC (*p* = 0.259) and nRMS (*p* = 0.114).

The pairwise comparisons between metrics are reported in Table 6. During Reaching, SPARC and NJ showed significantly lower nMAE than LDLJ (*p* < 0.001 and *p* = 0.015, respectively), while the other differences, including those between SPARC and NJ and between SPARC and nRMS, were not significant after correction. During Hand-to-mouth, all pairwise differences were significant (*p* ≤ 0.038), with nMAE increasing from SPARC to nRMS, NJ and LDLJ.

## 4. Discussion

This study investigated the validity of IMU-derived movement quality metrics against a marker-based motion capture system during two upper-limb tasks in healthy adults: Reaching and Hand-to-mouth. Specifically, SPARC, nRMS, NJ and LDLJ metrics were examined considering their linear relationship, estimation error, agreement, and proportional bias between the two measurement systems.

Overall, the results highlighted a clear task-dependent behavior, with a stronger correspondence between IMU- and MoCap-derived metrics during Reaching than during Hand-to-mouth.

In fact, the Reaching task showed the most consistent results across the four metrics. In particular, as shown in Table 1, significant positive correlations between IMU and MoCap measurements were observed for SPARC, nRMS, NJ, and LDLJ, with NJ showing the highest correlation coefficient. As regards agreement, the Bland–Altman analysis (Figure 3) showed that SPARC had a mean bias close to zero, with its 95% confidence interval including zero. nRMS also showed a small mean bias (−0.037), although its 95% confidence interval excluded zero, indicating a small but systematic difference between the two systems. More pronounced systematic differences between the two systems were observed for NJ and LDLJ. Moreover, significant proportional bias was identified for SPARC, NJ, and LDLJ (Table 2). For SPARC and LDLJ, increasing values of the metric were associated with progressively more negative MoCap-IMU differences, indicating a greater overestimation by the IMU at higher values. In contrast, for NJ, the difference between the two systems increased with the magnitude of the measurement, thus suggesting a greater underestimation by the IMU at higher values. Therefore, despite the significant correlations, the two systems cannot be considered interchangeable for all metrics and across the entire measurement range during the Reaching task.

The findings obtained during Reaching are consistent with previous literature supporting reaching movements as a suitable task for the quantitative assessment of upper-limb smoothness. In fact, reaching is one of the upper-limb movements on which the theoretical development and experimental evaluation of smoothness measures have largely been based [6]. Moreover, Refai et al. showed that SPARC was the smoothness metric that most consistently met the expected validity criteria during reach-to-point and reach-to-grasp tasks [4]. This result is consistent with the findings of the present study, in which SPARC showed a significant IMU-MoCap correlation and a mean bias close to zero during reaching. Furthermore, Cornec et al. showed that SPARC had high reliability and low measurement error during reaching after stroke. LDLJ showed similarly favorable results, whereas several temporal-domain smoothness measures were more affected by movement duration and noise [11], further highlighting the favorable measurement properties of these metrics during reaching movements.

In contrast, during the Hand-to-mouth task only SPARC and NJ showed significant positive correlations between IMU- and MoCap-derived values, whereas nRMS and LDLJ showed no significant linear relationship between the two systems. Furthermore, the 95% confidence intervals of the mean biases excluded zero for all four metrics. Particularly evident discrepancies were observed for NJ and LDLJ: the limits of agreement for NJ were entirely positive, indicating consistently lower values derived from IMU relative to MoCap within the observed agreement range; whereas those for LDLJ were entirely negative, indicating consistently higher IMU-derived values than MoCap. Moreover, as shown in Table 4, both SPARC and LDLJ showed significant proportional bias during the Hand-to-mouth task. Consistently with these findings, the RMSE and MAE were higher during Hand-to-mouth (Table 3) than during Reaching (Table 1) for all metrics, especially for NJ and LDLJ. Hence, considering together correlation, estimation error, and agreement analyses, our findings indicate that criterion validity was less consistent during Hand-to-mouth.

The comparison of normalized errors provided statistical support for these observations. The error increased significantly from Reaching to Hand-to-mouth for NJ and LDLJ, whereas it remained stable for SPARC and nRMS. Moreover, SPARC showed the lowest error in both tasks, and during Hand-to-mouth its error was significantly lower than that of all other metrics, while during Reaching it differed significantly only from LDLJ. These results identify SPARC as the most robust of the investigated metrics when derived from wrist-worn IMUs, consistently with its favorable measurement properties and lower dependence on movement duration reported in previous studies [4,10,11]. However, the significant proportional bias observed for SPARC in both tasks indicates that its relatively low estimation error does not imply full agreement between systems across the measurement range. Conversely, jerk-based metrics, and LDLJ in particular, appeared more sensitive to the differences between the two measurement systems as movement complexity increased.

This task dependence may partly reflect differences in movement complexity. Transporting the hand toward the mouth is biomechanically more complex than simple point-to-point reaching and involves a different combination of translational and rotational components of upper-limb movement. In fact, Murphy et al. divided a functional drinking task into distinct phases (reaching, forward-transport, drinking, backward-transport and returning to initial position) [7], highlighting that movement characteristics vary substantially from reaching a point to transport the hand to the mouth. Moreover, previous upper-limb kinematic studies have also treated Reaching and Hand-to-mouth as distinct motor tasks, reflecting their different movement organization and functional demands. In particular, Caimmi et al. assessed both tasks separately and quantified movement smoothness using NJ, further supporting the relevance of task-specific evaluation of upper-limb movement quality [23].

Another factor that may contribute to the observed task dependence is the different nature of the acceleration signals obtained from the two systems. Although both approaches provide acceleration-related information, they do not constitute identical physical measurements. IMU acceleration was measured directly by the sensor positioned at the wrist, whereas MoCap-derived acceleration was obtained by twice differentiating the position of the wrist marker over time. Therefore, discrepancies between the two signals may arise from the different measurement principles. Melendez-Calderon et al. showed that the estimation of movement smoothness from IMU-derived acceleration can be sensitive to the characteristics of the measured signal and to the processing approach adopted [14]. In the present study, the more complex kinematics of the Hand-to-mouth task may have amplified the differences between directly measured IMU acceleration and acceleration reconstructed from MoCap trajectories, thereby contributing to the lower agreement observed for this task.

A signal-level explanation for the lower agreement of nRMS, and partly of LDLJ, during Hand-to-mouth can be found in the treatment of gravity. In principle, the wrist-worn IMU and the wrist marker measure the linear acceleration of the same point, so components related to curved trajectories and segment rotation should be captured by both systems. However, since sensor orientation was not estimated, gravity was attenuated by subtracting the median acceleration vector within each repetition, which removes only its constant component. During Hand-to-mouth, elbow flexion and forearm rotation markedly change sensor orientation relative to gravity, leaving a time-varying gravitational residual in the IMU acceleration magnitude that has no counterpart in MoCap-derived acceleration. Amplitude-based metrics such as nRMS are directly affected by this residual. This suggests that the reduced agreement reflects the processing pipeline rather than an inherent limitation of the metric, and that orientation-based gravity compensation should be adopted in future applications.

A relevant comparison can be made with the study by Unger et al., who compared IMUs with MoCap in individuals after stroke during a functional drinking task and specifically calculated an acceleration-based LDLJ using IMU acceleration and acceleration obtained by double differentiation of the optical hand-marker position [19], an approach conceptually close to that adopted in the present study. Their results showed good agreement for LDLJ and for most of the investigated movement-quality measures. The difference from the present Hand-to-mouth findings is therefore noteworthy, but the studies are not equivalent in terms of IMU configuration, task segmentation and population. Rather than contradicting the present findings, this comparison emphasizes how strongly the validity of IMU-derived movement-quality metrics depends on the complete measurement and processing pipeline. A similar methodological dependence has been reported in other IMU validation studies. Merlau et al. compared IMU- and MoCap-derived upper-limb movement parameters during reach and grasp tasks after stroke and showed that agreement was strongly affected by calibration and data-processing procedures [18]. Systematic reviews have highlighted considerable heterogeneity in sensor placement, algorithms, movement protocols, and outcome definitions across studies investigating upper-limb kinematics and wearable-sensor-based [2,24]. Accordingly, the present findings should not be interpreted as generalizable evidence of validity for these IMU-derived metrics. They should instead be considered specific to the experimental protocol adopted here, including the selected tasks, wrist sensor placement, acceleration-based processing, and MoCap reference method. Further validation is required before extending these results to other tasks, sensor configurations, processing procedures, or populations.

Regarding NJ, the IMU-MoCap correlation was r = 0.741 in Reaching and r = 0.390 in Hand-to-mouth, but the mean bias was large (3.384 and 6.904, respectively), with entirely positive limits of agreement during Hand-to-mouth. This indicates that the two systems rank participants similarly but express NJ on different absolute scales. Therefore, IMU-derived NJ should not be interpreted as an absolute smoothness value, nor compared with MoCap-derived values or reference thresholds. At most, it could be used as a relative index, for example to follow changes over time using the same device, task and processing pipeline.

Given the present level of validity, wrist-worn IMUs cannot yet be considered a direct replacement for MoCap, which would require an accurate classification of individuals against reference values. A more reasonable role is that of a relative index for longitudinal follow-up, in which changes are tracked within the same individual using the same device, metric and task. In this setting, a constant bias between systems cancels out, whereas a proportional bias, as observed for SPARC and LDLJ in both tasks and for NJ during Reaching, affects the magnitude but not the direction of the detected changes. Given its lower normalized error and stability across tasks, SPARC appears the most suitable candidate for this purpose. However, the reliability and responsiveness of these metrics were not assessed here and should be confirmed in clinical populations.

Some limitations should be considered. First, the study was conducted in young healthy adults; hence, the present results cannot be directly generalized to neurological populations. Previous studies have demonstrated the clinical relevance of upper-limb kinematics and smoothness after stroke [9,25], but validation in pathological movement remains necessary before clinical application. Pathological movements are typically slower, more segmented and more variable than those of healthy individuals, which may affect the agreement between systems differently from what was observed here. Future studies should therefore assess agreement in individuals after stroke and compare it with that obtained in healthy participants. Moreover, the limited between-subject variability of movement smoothness in healthy young adults may have reduced the magnitude of the observed correlations. Second, only two specific upper-limb tasks were examined, and the clear differences observed between them indicate that validity is task-dependent. Third, gravity was not compensated using sensor orientation, and the sensitivity of the results to this choice should be assessed in future studies using sensor-fusion-based gravity removal. Additional functional tasks should therefore be evaluated before generalizing these findings to other movement conditions.

## 5. Conclusions

The present study showed that wrist-worn IMUs can provide movement-quality metrics that reflect MoCap-derived measurements, although their criterion validity was clearly both metric- and task-dependent. Overall, the Reaching task showed the most favorable results: all four metrics showed significant IMU-MoCap correlations, with SPARC showing the closest average agreement, as its mean bias did not significantly differ from zero. Taken together, our findings suggest that, within the present experimental protocol, IMU-derived metrics are more consistently supported for Reaching than for Hand-to-mouth. Among the investigated metrics, SPARC showed the most consistent behavior across tasks, whereas NJ also captured between-subject variations in both tasks but with large systematic differences between systems, so that its absolute values are not interchangeable with MoCap-derived ones. In contrast, nRMS and especially LDLJ showed greater task dependence, with their validity being less supported during Hand-to-mouth.

Rather than replacing MoCap, wrist-worn IMU-derived metrics, and SPARC in particular, may be most appropriately used, rather than absolute measures of smoothness, as relative indices for longitudinal follow-up, provided that their reliability and responsiveness are confirmed in clinical populations.

## Figures and Tables

**Table 1:** Pearson correlation coefficients (r) with 95% confidence intervals (CI), associated *p*-values, root mean square error (RMSE), and mean absolute error (MAE) for the comparison between IMU- and MoCap-derived metrics during the Reaching task.

Index	Pearson r [95% CI]	*p*	RMSE	MAE
SPARC	0.491 [0.193, 0.668]	<0.001	0.4121	0.3252
nRMS	0.412 [0.125, 0.620]	0.002	0.0454	0.0388
NJ	0.741 [0.498, 0.885]	<0.001	3.8580	3.4092
LDLJ	0.505 [0.315, 0.672]	<0.001	0.5874	0.4851

**Table 2:** Bland–Altman agreement and proportional bias results for the comparison between IMU- and MoCap-derived metrics during the Reaching task. Mean bias is expressed as the MoCap-IMU difference with its 95% confidence interval (CI), together with the 95% limits of agreement (LoA). Slope B represents the regression coefficient of the paired differences against the mean of the two measurements and was used to assess proportional bias; *p* < 0.05 indicates the presence of a significant proportional bias.

Index	Mean Bias [95% CI]	95% LoA [Lower, Upper]	Slope B [95% CI]	*p*
SPARC	0.046 [−0.032, 0.128]	[−0.764, 0.856]	−0.329 [−0.713, −0.116]	<0.001
nRMS	−0.037 [−0.045, −0.029]	[−0.088, 0.014]	0.208 [−0.185, 0.559]	0.262
NJ	3.384 [2.777, 4.078]	[−0.280, 7.049]	0.328 [0.064, 0.521]	0.013
LDLJ	−0.482 [−0.592, −0.375]	[−1.146, 0.182]	−0.546 [−0.816, −0.220]	<0.001

**Table 3:** Pearson correlation coefficients (r) with 95% confidence intervals (CI), associated *p*-values, root mean square error (RMSE), and mean absolute error (MAE) for the comparison between IMU- and MoCap-derived metrics during the Hand-to-mouth task.

Index	Pearson r [95% CI]	*p*	RMSE	MAE
SPARC	0.466 [0.192, 0.663]	<0.001	0.4347	0.3623
nRMS	0.150 [−0.142, 0.408]	0.278	0.0508	0.0434
NJ	0.390 [0.107, 0.613]	0.006	7.1757	6.9043
LDLJ	−0.047 [−0.471, 0.328]	0.740	1.0924	1.0048

**Table 4:** Bland–Altman agreement and proportional bias results for the comparison between IMU- and MoCap-derived metrics during the Hand-to-mouth task. Mean bias is expressed as the MoCap-IMU difference with its 95% confidence interval (CI), together with the 95% limits of agreement (LoA). Slope B represents the regression coefficient of the paired differences against the mean of the two measurements and was used to assess proportional bias; *p* < 0.05 indicates the presence of a significant proportional bias.

Index	Mean Bias [95% CI]	95% LoA [Lower, Upper]	Slope B [95% CI]	*p*
SPARC	0.233 [0.134, 0.333]	[−0.494, 0.959]	−0.328 [−0.648, 0.037]	0.028
nRMS	−0.041 [−0.051, −0.032]	[−0.100, 0.017]	−0.448 [−0.925, 0.004]	0.052
NJ	6.904 [6.245, 7.512]	[3.033, 10.775]	0.108 [−0.344, 0.502]	0.600
LDLJ	−1.005 [−1.152, −0.862]	[−1.853, −0.157]	−1.244 [−1.903, −0.854]	<0.001

**Table 5:** Normalized mean absolute error (nMAE) of IMU-derived metrics with respect to MoCap for the Reaching and Hand-to-mouth tasks, and difference between tasks. Values are reported with 95% confidence intervals (CI); *p*-values are Holm-adjusted.

Index	Reaching nMAE [95% CI]	Hand-to-Mouth nMAE [95% CI]	Difference H2M—Reaching [95% CI]	*p*
SPARC	0.93 [0.70, 1.32]	1.17 [0.91, 1.62]	0.25 [−0.18, 0.65]	0.259
nRMS	1.52 [1.16, 2.20]	2.22 [1.79, 2.93]	0.70 [−0.02, 1.43]	0.114
NJ	1.23 [0.99, 1.64]	3.73 [2.95, 5.14]	2.50 [1.70, 3.83]	<0.001
LDLJ	1.92 [1.46, 2.53]	5.28 [4.11, 7.18]	3.37 [2.08, 5.29]	<0.001

**Table 6:** Pairwise comparisons of nMAE between metrics within each task. The difference is computed as first minus second metric; negative values indicate lower error for the first metric. Values are reported with 95% confidence intervals (CI), not adjusted for multiple comparisons; *p*-values are Holm-adjusted.

Task	Comparison	Difference [95% CI]	*p*
Reaching	SPARC vs. nRMS	−0.59 [−1.23, −0.04]	0.141
	SPARC vs. NJ	−0.30 [−0.77, 0.16]	0.715
	SPARC vs. LDLJ	−0.99 [−1.64, −0.39]	<0.001
	nRMS vs. NJ	0.29 [−0.26, 1.01]	0.715
	nRMS vs. LDLJ	−0.40 [−1.20, 0.54]	0.715
	NJ vs. LDLJ	−0.69 [−1.17, −0.23]	0.015
Hand-to-mouth	SPARC vs. nRMS	−1.05 [−1.76, −0.47]	0.007
	SPARC vs. NJ	−2.56 [−3.99, −1.59]	<0.001
	SPARC vs. LDLJ	−4.11 [−5.86, −2.96]	<0.001
	nRMS vs. NJ	−1.51 [−3.06, −0.37]	0.021
	nRMS vs. LDLJ	−3.06 [−5.02, −1.72]	<0.001
	NJ vs. LDLJ	−1.56 [−3.10, −0.09]	0.038

## Data Availability

Data are available from the corresponding author upon reasonable request.

## Abbreviations

The following abbreviations are used in this manuscript:

IMU	Inertial Measurement Unit
MoCap	Marler-based Motion Capture system
SPARC	Spectral Arc Length
nRMS	Normalized Root Mean Square
NJ	Normalized Jerk
LDLJ	Log Dimensionless Jerk

## References

[1] Santisteban L., Térémetz M., Bleton J.-P., Baron J.-C., Maier M.A., Lindberg P.G. (2016). Upper Limb Outcome Measures Used in Stroke Rehabilitation Studies: A Systematic Literature Review. PLoS ONE.

[2] Schwarz A., Kanzler C.M., Lambercy O., Luft A.R., Veerbeek J.M. (2019). Systematic Review on Kinematic Assessments of Upper Limb Movements After Stroke. Stroke.

[3] Kwakkel G., Van Wegen E., Burridge J., Winstein C., Van Dokkum L., Alt Murphy M., Levin M., Krakauer J. (2019). Standardized Measurement of Quality of Upper Limb Movement after Stroke: Consensus-Based Core Recommendations from the Second Stroke Recovery and Rehabilitation Roundtable. Int. J. Stroke.

[4] Mohamed Refai M.I., Saes M., Scheltinga B.L., Van Kordelaar J., Bussmann J.B.J., Veltink P.H., Buurke J.H., Meskers C.G.M., Van Wegen E.E.H., Kwakkel G. (2021). Smoothness Metrics for Reaching Performance after Stroke. Part 1: Which One to Choose?. J. Neuroeng. Rehabil..

[5] Rohrer B., Fasoli S., Krebs H.I., Hughes R., Volpe B., Frontera W.R., Stein J., Hogan N. (2002). Movement Smoothness Changes during Stroke Recovery. J. Neurosci..

[6] Balasubramanian S., Melendez-Calderon A., Roby-Brami A., Burdet E. (2015). On the Analysis of Movement Smoothness. J. Neuroeng. Rehabil..

[7] Murphy M.A., Willén C., Sunnerhagen K.S. (2011). Kinematic Variables Quantifying Upper-Extremity Performance After Stroke During Reaching and Drinking From a Glass. Neurorehabil. Neural Repair.

[8] Alt Murphy M., Willén C., Sunnerhagen K.S. (2013). Responsiveness of Upper Extremity Kinematic Measures and Clinical Improvement During the First Three Months After Stroke. Neurorehabil. Neural Repair.

[9] Collins K.C., Kennedy N.C., Clark A., Pomeroy V.M. (2018). Kinematic Components of the Reach-to-Target Movement After Stroke for Focused Rehabilitation Interventions: Systematic Review and Meta-Analysis. Front. Neurol..

[10] Bayle N., Lempereur M., Hutin E., Motavasseli D., Remy-Neris O., Gracies J.-M., Cornec G. (2023). Comparison of Various Smoothness Metrics for Upper Limb Movements in Middle-Aged Healthy Subjects. Sensors.

[11] Cornec G., Lempereur M., Mensah-Gourmel J., Robertson J., Miramand L., Medee B., Bellaiche S., Gross R., Gracies J.-M., Remy-Neris O. (2024). Measurement Properties of Movement Smoothness Metrics for Upper Limb Reaching Movements in People with Moderate to Severe Subacute Stroke. J. Neuroeng. Rehabil..

[12] Maceira-Elvira P., Popa T., Schmid A.-C., Hummel F.C. (2019). Wearable Technology in Stroke Rehabilitation: Towards Improved Diagnosis and Treatment of Upper-Limb Motor Impairment. J. Neuroeng. Rehabil..

[13] Kim G.J., Parnandi A., Eva S., Schambra H. (2022). The Use of Wearable Sensors to Assess and Treat the Upper Extremity after Stroke: A Scoping Review. Disabil. Rehabil..

[14] Melendez-Calderon A., Shirota C., Balasubramanian S. (2021). Estimating Movement Smoothness From Inertial Measurement Units. Front. Bioeng. Biotechnol..

[15] Giavarina D. (2015). Understanding Bland Altman Analysis. Biochem. Med..

[16] Akoglu H. (2018). User’s Guide to Correlation Coefficients. Turk. J. Emerg. Med..

[17] Hughes C.M.L., Louie A., Sun S., Gordon-Murer C., Belay G.J., Baye M., Zhang X. (2019). Development of a Post-Stroke Upper Limb Rehabilitation Wearable Sensor for Use in Sub-Saharan Africa: A Pilot Validation Study. Front. Bioeng. Biotechnol..

[18] Merlau B., Cormier C., Alaux A., Morin M., Montané E., Amarantini D., Gasq D. (2023). Assessing Spatiotemporal and Quality Alterations in Paretic Upper Limb Movements after Stroke in Routine Care: Proposal and Validation of a Protocol Using IMUs versus MoCap. Sensors.

[19] Unger T., De Sousa Ribeiro R., Mokni M., Weikert T., Pohl J., Schwarz A., Held J.P.O., Sauerzopf L., Kühnis B., Gavagnin E. (2024). Upper Limb Movement Quality Measures: Comparing IMUs and Optical Motion Capture in Stroke Patients Performing a Drinking Task. Front. Digit. Health.

[20] Kristianslund E., Krosshaug T., Van Den Bogert A.J. (2012). Effect of Low Pass Filtering on Joint Moments from Inverse Dynamics: Implications for Injury Prevention. J. Biomech..

[21] Flash T., Hogan N. (1985). The Coordination of Arm Movements: An Experimentally Confirmed Mathematical Model. J. Neurosci..

[22] Hogan N., Sternad D. (2009). Sensitivity of Smoothness Measures to Movement Duration, Amplitude, and Arrests. J. Mot. Behav..

[23] Caimmi M., Guanziroli E., Malosio M., Pedrocchi N., Vicentini F., Molinari Tosatti L., Molteni F. (2015). Normative Data for an Instrumental Assessment of the Upper-Limb Functionality. BioMed Res. Int..

[24] Martino Cinnera A., Picerno P., Bisirri A., Koch G., Morone G., Vannozzi G. (2024). Upper Limb Assessment with Inertial Measurement Units According to the International Classification of Functioning in Stroke: A Systematic Review and Correlation Meta-Analysis. Top. Stroke Rehabil..

[25] Saes M., Mohamed Refai M.I., Van Beijnum B.J.F., Bussmann J.B.J., Jansma E.P., Veltink P.H., Buurke J.H., Van Wegen E.E.H., Meskers C.G.M., Krakauer J.W. (2022). Quantifying Quality of Reaching Movements Longitudinally Post-Stroke: A Systematic Review. Neurorehabil. Neural Repair.

